# A systematic meta-analysis of oxygen-to-glucose and oxygen-to-carbohydrate ratios in the resting human brain

**DOI:** 10.1371/journal.pone.0204242

**Published:** 2018-09-24

**Authors:** Tyler Blazey, Abraham Z. Snyder, Manu S. Goyal, Andrei G. Vlassenko, Marcus E. Raichle

**Affiliations:** 1 Mallinckrodt Institute of Radiology, Washington University School of Medicine, St. Louis, MO, United States of America; 2 Department of Neurology, Washington University School of Medicine, St. Louis, MO, United States of America; 3 Department of Biomedical Engineering, Washington University, St. Louis, MO, United States of America; Taipei Veterans General Hospital, TAIWAN

## Abstract

Glucose is the predominant fuel supporting brain function. If the brain’s entire glucose supply is consumed by oxidative phosphorylation, the molar ratio of oxygen to glucose consumption (OGI) is equal to 6. An OGI of less than 6 is evidence of non-oxidative glucose metabolism. Several studies have reported that the OGI in the resting human brain is less than 6.0, but the exact value remains uncertain. Additionally, it is not clear if lactate efflux accounts for the difference between OGI and its theoretical value of 6.0. To address these issues, we conducted a meta-analysis of OGI and oxygen-to-carbohydrate (glucose + 0.5*lactate; OCI) ratios in healthy young and middle-aged adults. We identified 47 studies that measured at least one of these ratios using arterio-venous differences of glucose, lactate, and oxygen. Using a Bayesian random effects model, the population median OGI was 5.46 95% credible interval (5.25–5.66), indicating that approximately 9% of the brain’s glucose metabolism is non-oxidative. The population median OCI was 5.60 (5.36–5.84), suggesting that lactate efflux does not account for all non-oxidative glucose consumption. Significant heterogeneity across studies was observed, which implies that further work is needed to characterize how demographic and methodological factors influence measured cerebral metabolic ratios.

## Introduction

Glucose and oxygen consumption are tightly coupled in the brain at rest, with the majority of glucose undergoing complete oxidative phosphorlyation[1]. Furthermore, the ratio of carbon dioxide production to oxygen consumption is very close to one[2], indicating that nearly all of oxygen consumption is used for carbohydrates. The standard measure of coupling between oxygen and glucose utilization is the oxygen-to-glucose index (OGI), which is the molar ratio of oxygen to glucose consumption. An OGI of 6 indicates that all glucose is consumed via oxidative pathways.

The measurement of cerebral arterio-venous differences of oxygen and glucose is regarded as the gold-standard technique for obtaining OGI. With this method, arterial samples are collected from a peripheral artery (e.g. radial or brachial artery) and venous samples from the internal jugular vein at the jugular bulb. The primary assumption of this technique is that the venous blood in the jugular bulb comes solely from the brain. If blood from other sources is present, than the arterio-venous difference is no longer only the result of cerebral metabolism. This bias is likely to be small, however, as it has been estimated that 97.4% of the blood in the jugular bulb comes from cerebral sources[3].

Although the arterio-venous technique has been used to study whole-brain OGI for over sixty years[4], there remains some uncertainty as to the exact value. Individual studies using arterio-venous differences in humans at rest have reported values ranging from 4.6[5] to 7.5[6]. In 1957, Kety reviewed sixteen studies of both healthy and diseased populations and reported a mean value of 5.54[4]. A more recent meta-analysis of eight studies of metabolism during exercise found a whole-brain OGI of 5.1[7]. These two reviews suggest that anywhere from 8 to 15% of the brain’s glucose uptake is consumed via non-oxidative metabolism. Thus, the value of cerebral OGI in resting, healthy humans is known only approximately.

The fate of glucose consumed by non-oxidative pathways is also a matter of some debate. It has been suggested that lactate efflux to venous blood may completely account for non-oxidative glucose metabolism[8]. Two more recent reviews have reported conflicting results[7,9]. Both studies performed a meta-analysis of the oxygen-to-carbohydrate index (OCI), also referred to as the cerebral metabolic ratio (CMR). The OCI is computed as the molar ratio of the arterio-venous difference of oxygen to glucose plus ½ lactate. (The factor of ½ arises because each mole of glucose theoretically yields two moles of lactate). If lactate efflux to venous blood completely accounts for an OGI less than 6, then the OCI should equal 6 or greater. Alternatively, an OCI less than 6 indicates that lactate efflux to venous blood does not alone account for all of non-oxidative glucose metabolism. Consistent with the original finding of Siesjö[8], Quistroff et al. reported that the population mean OCI from eight studies is approximately 6. However, Rasmussen et al., in a partially overlapping sample of eight studies, reported that the resting OCI was 5.3. Thus, it remains unclear whether lactate fully accounts for non-oxidative glucose metabolism in the resting human brain.

To provide a more accurate estimate of both OGI and OCI in the healthy human brain at rest, we conducted a systematic meta-analysis[10] of studies reporting arterio-venous differences for glucose, oxygen, and lactate. We identified 40 studies with OGI data and 37 partially overlapping studies with OCI data. We then performed a random effects Bayesian meta-analysis[11] to determine the population average OGI and OCI ratios and their credible intervals (CI s).

## Results

### Included studies

**Fig 1:**
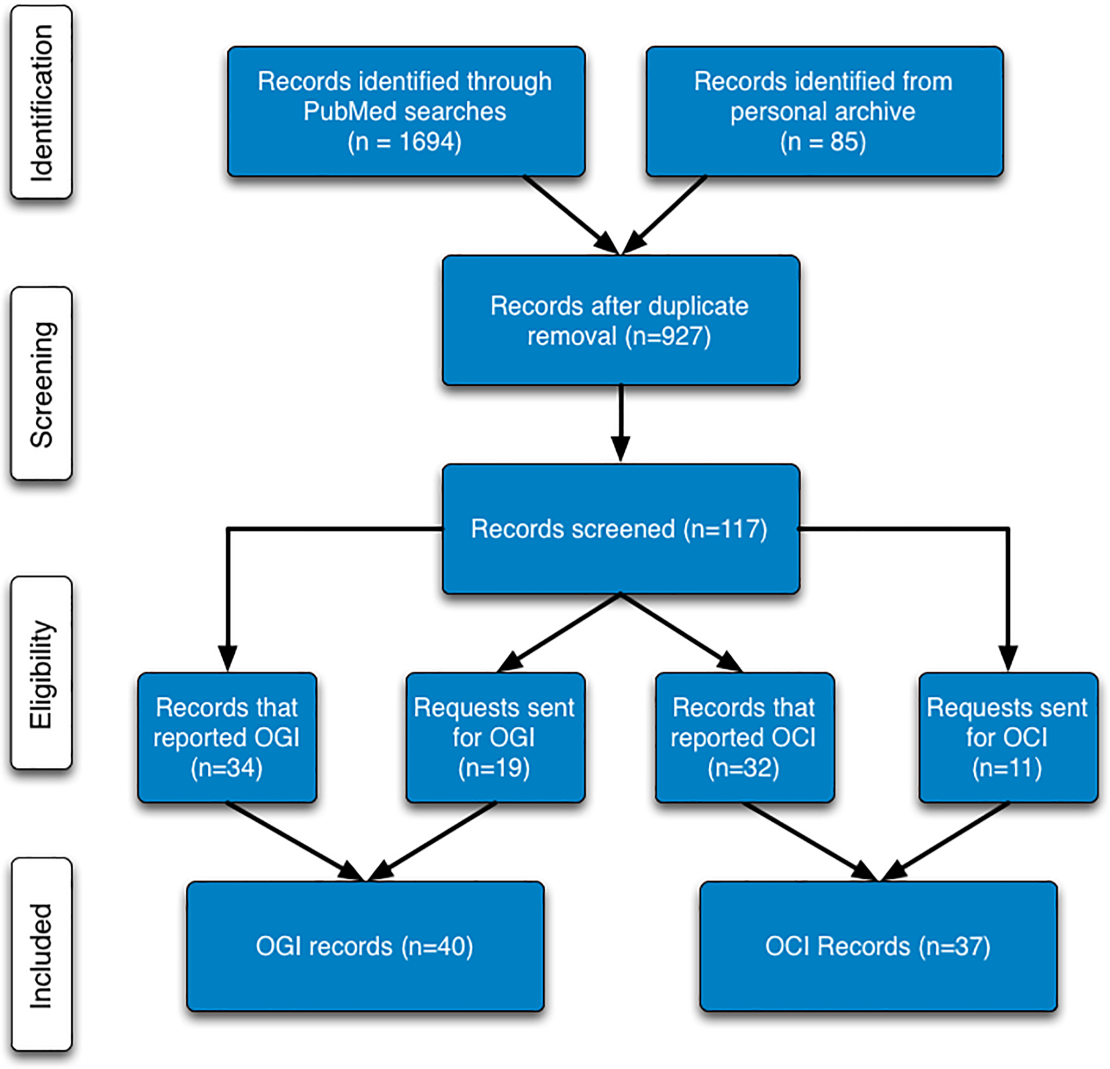
Modified PRISMA flow diagram. Included studies were selected using the indicated selection criteria.

Our searches of PUBMED (see methods) and our own archives identified 927 potential records (Fig 1). After reviewing the titles, and if necessary, abstracts of all 927 records, 810 were discarded from further consideration. Records were discarded at this step if they were clearly irrelevant for our purposes (e.g. animal studies). The remaining 117 papers were then subjected to a critical full text review. This review resulted in the rejection of 65 papers (S1 Table). The majority of papers were rejected because they did not acquire the data necessary to calculate OGI/OCI (n = 38) or because they reported values only in experimental states (n = 17). For OGI, we found 52 papers that met our requirements for inclusion, 34 of which reported OGI. In addition, we sent 19 requests for data to authors of studies that had the data necessary to report OGI but did not do so. We received data from 6 of these authors, resulting in a total of 40 studies. For OCI, 43 papers met our inclusion requirements. Of these, 32 papers reported the required data, and data requests were sent for the remaining 11. After receiving data from 5 authors, our final OCI dataset contained 37 studies. A summary of the characteristics for the included studies is in S2 Table. A total of 30 studies measured both OGI and OCI.

### Population average OGI and OCI

**Fig 2:**
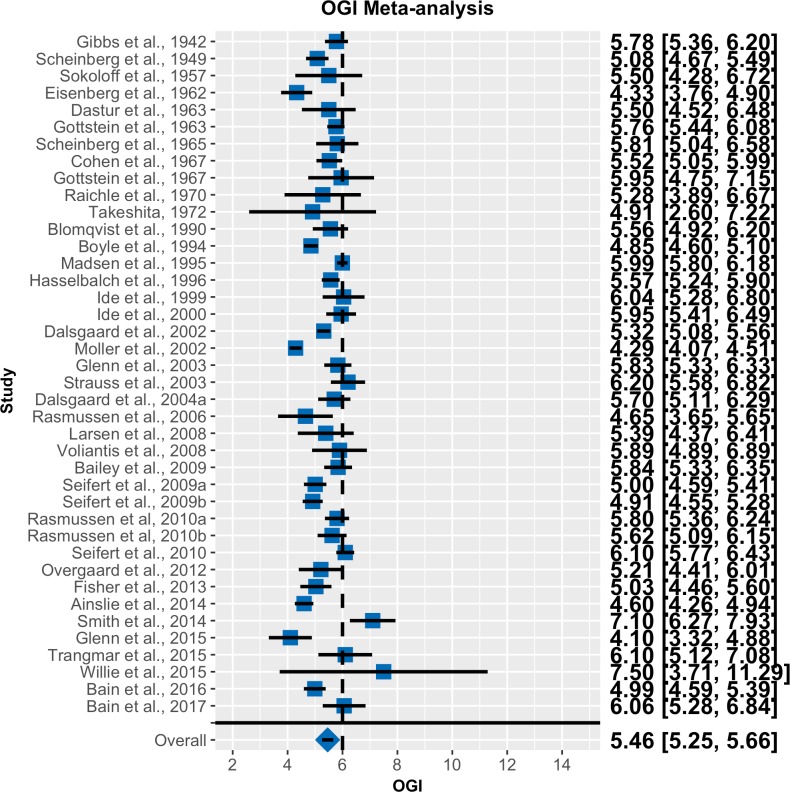
Forest plot for OGI meta-analysis. Blue squares represent the reported mean OGI for each study. Black lines represent 95% confidence intervals. Numeric values for these quantities are also listed. The blue diamond is the population average from the Bayesian random effects meta-analysis. Error bars/values for the population mean are 95% CIs (n = 40).

**Fig 3:**
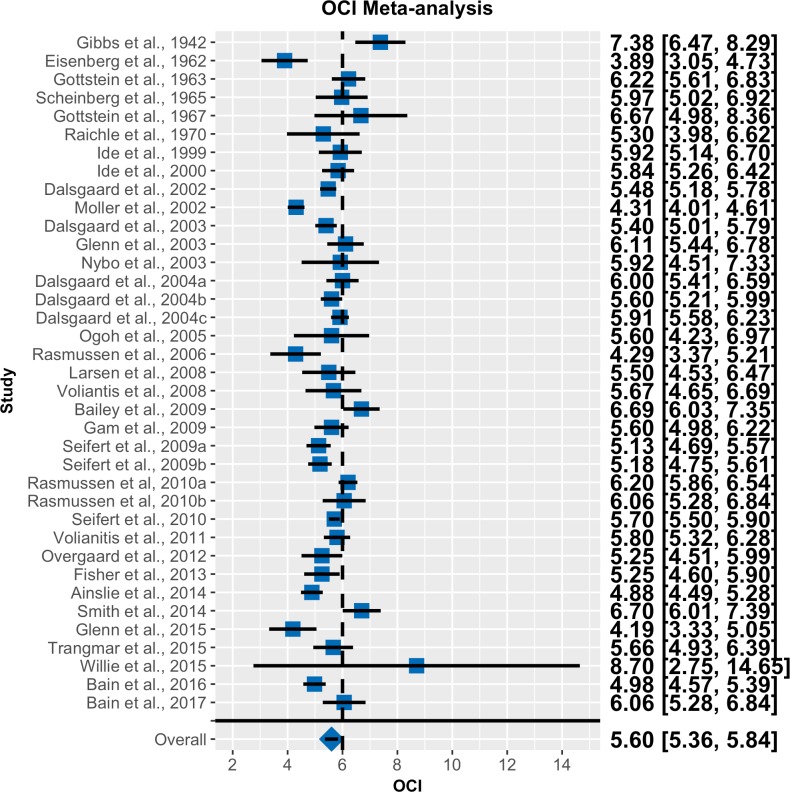
Forest plot for OCI meta-analysis. Same convention as in Fig 2 (n = 37).

Forest plots for OGI and OCI are shown in Figs 2 and 3, respectively. Note that the random effects models effectively decrease the weight of studies with high standard errors. The population average OGI was 5.46 with a 95% CI of 5.25 to 5.66. As the CI does not overlap 6.0, we can infer that there is significant non-oxidative glucose consumption at rest. The population average OCI was 5.60 with a 95% CI of 5.36 to 5.84. The fact that the credible intervals do not contain 6 indicates that a significant portion of the brain’s glucose consumption is non-oxidative and cannot be accounted for by lactate efflux to the blood.

### Bias and heterogeneity

Within-study bias was assessed in four separate categories: study population, waiting period between catheterization and measurement, experimental manipulations, and fasting state (S3 Table). The most frequent bias in study population was the use of all male subjects. Nineteen studies included only male subjects. No study included only female subjects. The majority of studies consisted of younger subjects (S3 Table). Across all studies that reported an average age, the mean age was 27.2 with a standard deviation (SD) of 4.6. Only five studies specifically mentioned including subjects over the age of 40[12–16]. A few other studies included only hospital patients (e.g. Scheinberg et al., 1949, Takeshita et al., 1972) or competitive athletes (e.g., Voliantis et al., 2008 and Bain et al., 2016). More than half (24/47) of studies included no mention of a waiting period between catheterization and blood sampling. Blood sampling was performed in a variety of positions, the two most common being supine (13) and semi-supine (20). The majority of measurements were performed in the absence of any overt experimental manipulation, however a few studies did include the injection of labeled compounds (e.g., Boyle et al., 1994 and Glenn et al., 2015) or saline (Hasselbalch et al., 1996 and Volianitis et al., 2011). Finally, the requirement for fasting subjects was mixed, with 19 requiring at least some fasting period, 20 including no mention of performing measurements in a fasting state, and the remaining 8 studies assessed subjects in a post-absorptive state.

**Fig 4:**
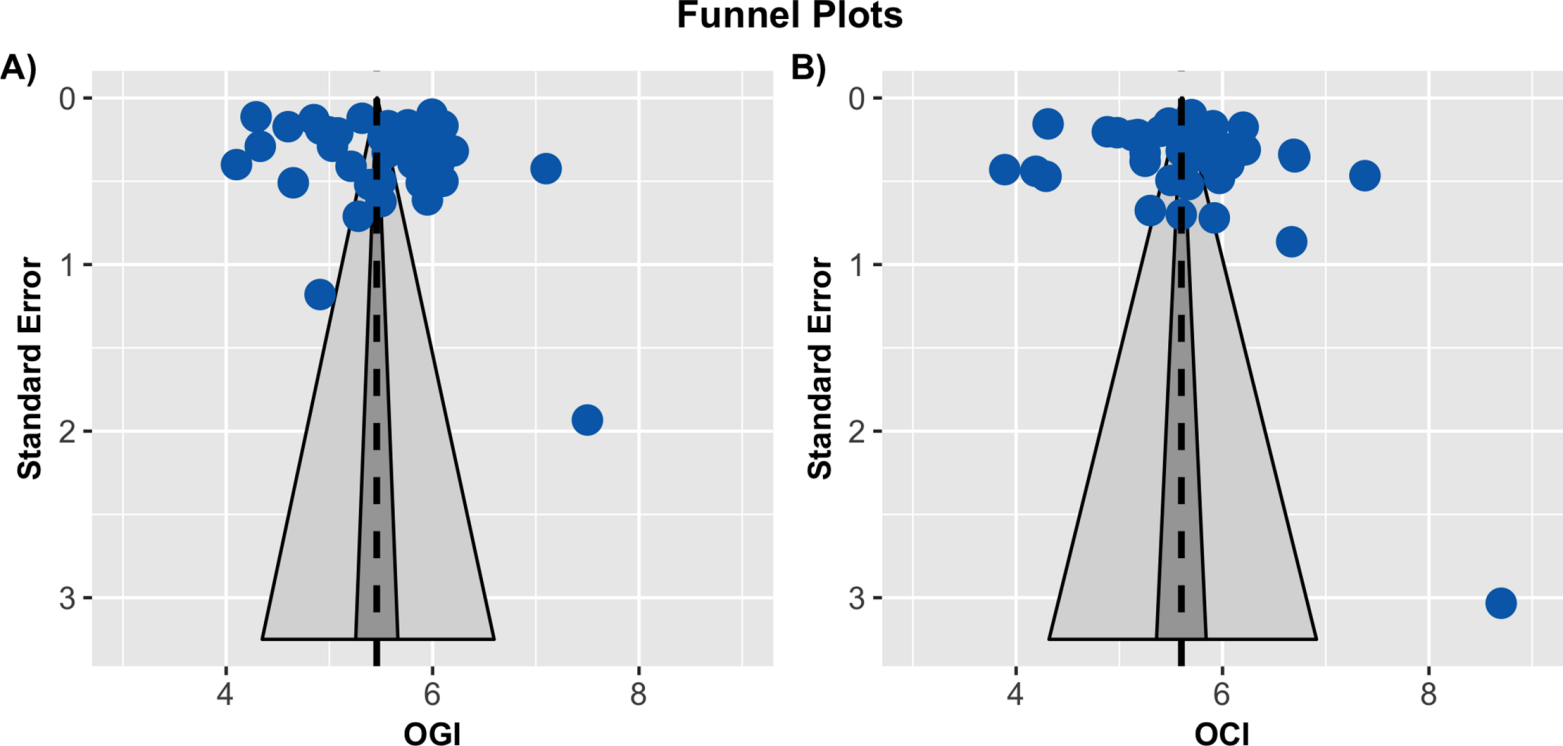
**Funnel plots for OGI (A) and OCI (B)**. In each plot, the reported study mean is plotted against its standard error. The population average is the dashed black line, its 95% percent CI is in dark gray, and its 95% prediction interval is in light gray. The lack of any asymmetry is evidence against substantial publication bias. The wide scatter around the population average, however, suggests that there is substantial heterogeneity between studies.

To assess bias across studies, funnel plots were constructed for both OGI (Fig 4A) and OCI (Fig 4B). No asymmetry was apparent in either plot. This impression was quantified with a regression test for asymmetry[17]. No significant evidence for asymmetry was found for either OGI (*p* = 0.2013) or OCI (*p* = 0.1948). The lack of asymmetry suggests the absence of reporting bias in our sample. There was, however, substantial horizontal scatter around the population averages, indicating heterogeneity across studies. To further assess this heterogeneity, we computed posterior predictive intervals for a new random study for each ratio. Both ratios showed considerable variability, with the 95% posterior predictive interval for OGI spanning 4.35 to 6.60 and from 4.32 to 6.91 for OCI. Furthermore, the *I*^2^ values were consistent with substantial between study heterogeneity. An estimated 85.03% [95 CI 75.88–91.35] of the total variance in the OGI meta-analysis was due to study heterogeneity. A similar value of 84.96% [95 CI 75.09–91.60] was found in the OCI analysis.

## Discussion

Our meta-analyses of OGI and OCI reveals that both measures are significantly less than 6. The fact that OGI is less than 6 indicates that a proportion of glucose consumption is non-oxidative, while OCI being less than 6 shows that not all of non-oxidative metabolism can be accounted for by lactate efflux to venous blood. Expressed in terms of percentages non-oxidative metabolism accounts for 9.0% 95 CI [5.67–12.5] and of glucose consumption and 6.7% 95 CI [2.67–10.67] of carbohydrate metabolism Our estimates of the population average OGI (5.46 95% CI [5.25–5.66], and OCI (5.60 with a 95% CI [5.36–5.84]) are based on a much larger set of studies than previous reviews, and are therefore more likely to accurately reflect the true population means. It is of some interest to note the close agreement between our population average OGI and the value of 5.54 originally reported by Kety[4].

Although we did not find any evidence for publication bias, we did find considerable heterogeneity across studies. We computed *I*^2^ for each ratio, which indicated that ~85% of the total variance is attributable to study heterogeneity. Substantial methodical differences (S4 Table) may account for the variability in measured OGI and OCI values. Many studies included only males and there is evidence of differences in metabolism between males and females[18]. Thus, it is likely that our population averages are more representative of male metabolic ratios. Similarly, our population averages are weighted towards the predominantly young adult samples included in our meta-analysis. Many studies also did not specify if they included a waiting period between catheterization and measurement. This may have influenced the reported values, as metabolic ratios have been shown to decrease during arousal[19]. Finally, not all investigators insured that measurements were performed while subjects were in a basal metabolic state. A few studies infused labeled carbohydrates, and many studies did require that subjects be in a fasting state. Either factor could have affected the published results. For example, OGI is known to increase during hypoglycemia[16]. More direct studies are clearly needed to quantify the sources of heterogeneity in studies measuring OGI and OCI.

There is no clear consensus concerning the role of non-oxidative glucose metabolism in the brain[20]. It has been variously proposed that non-oxidative glucose consumption (i) allows for the rapid creation of ATP for the Na^+^/K^+^ ATPase in astrocytes[21], (ii) regulates cellular redox potentials[22], (iii) is a by-product of glycogen breakdown during increased neuronal activation[23], (iv) is necessary for the degradation of glutamate by astrocytes[24], (v) reduces oxidative stress, particularly during periods of cellular growth[25], or (vi) is used to fuel biosynthetic processes[26,27]. Part of the difficulty here is the uncertainty regarding the ultimate fate of glucose that enters non-oxidative pathways. It was traditionally thought that lactate production, and subsequent efflux to venous blood, could completely account for any non-oxidative glucose use[8]. The results of our meta-analysis are not consistent with this idea. The fact that the population average OCI was greater than the average OGI does show that some glucose is converted to lactate and leaves the brain via the venous system. The OCI was less than 6, however, which means this route does not account for all non-oxidative glucose use.

One potential explanation for the OCI being less than 6 is that resting arterio-venous differences simply underestimate the amount of lactate that leaves the brain. Brain lactate concentration has been shown to decrease during sleep[28], suggesting that measurements taken during conscious rest do not fully account for all of lactate efflux. Alternatively, lactate may leave the brain via routes that bypass the sampling sites used for arterio-venous differences. This idea is supported by a study by Ball et al., who found that injection of radiolabeled glucose and lactate into the inferior colliculus labeled the meninges[29]. Subsequent tracer experiments identified a potential perivascular clearance pathway from the inferior colliculus to the cervical lymph nodes[29]. More recently, components of the glymphatic system have been shown in mice to regulate lactate efflux as well as the concentration of lactate in cervical lymph nodes[28]. Neither of these experiments, however, quantified the proportion of lactate efflux that occurs via these pathways. Furthermore, if perivascular/glymphatic clearance does play a role in lactate removal, it is not clear what impact it would have on arterio-venous difference measurements. In sheep, rats, and rabbits approximately half of CSF is cleared through lymphatic pathways[30]. The other half enters the venous sinuses through the arachnoid villi, and therefore would presumably be accounted for by venous samples taken at the level of the jugular bulb. Although exact proportions are not available, it has been proposed that the arachnoid pathway plays a much a larger role in humans[30]. If true, this would suggest that perivascular/glymphatic clearance cannot fully account for the OCI being less than 6. Direct experimental approaches are clearly needed to address this question.

An alternative possibility is that the carbon from non-oxidative glucose metabolism leaves the brain as metabolites other than CO_2_ or lactate. Although pyruvate is well-known to have a net efflux from the brain, it is unlikely to account for much of the unexplained fraction, as net pyruvate efflux is nearly an order of magnitude less than that of lactate[31]. Numerous other carbon-containing compounds, however, have also been shown to leave the brain. For example, there is a small net efflux of glutamine from the brain[32,33]. In addition, peptides and proteins are known to exit the brain via the CSF[34]. The most well-studied of these are amyloid-beta[35,36] and tau[37], which are both markers of Alzheimer’s disease[38]. Other molecules, such as leptin[39] and cholesterol[40], have also been shown to leave the brain in small amounts. Future experiments with labeled compounds are needed to elucidate how, and in what proportions, glucose derived carbon leaves the brain.

Although we are not aware of any studies directly linking non-oxidative glucose consumption with the synthesis, and subsequence efflux, of specific glucose metabolites, there is evidence linking non-oxidative metabolism with biosynthesis more generally. Madsen et al., found that OGI was depressed after the performance of the Wisconsin Card Sorting task, while lactate efflux returned to baseline values[41]. Similarly, our group recently reported that, hours after the performance of a covert motor learning task, non-oxidative glucose use was elevated in Brodmann Area 44[42]. Moreover, the change in non-oxidative glucose use was positively correlated with performance during the learning task. Both of these studies are consistent with the hypothesis that glucose is used in a non-oxidative manner to support learning-induced synaptic plasticity. Extending these findings to other learning paradigms (e.g. episodic memory) would provide additional evidence along these lines.

A prior meta-analysis from our group found that non-oxidative glucose use is markedly elevated during early childhood[27], a period of brain growth[43]. This finding was recently supported by Segarra-Mondejar et al., who found that glucose consumption is necessary for neurite outgrowth *in vitro* and *in vivo* [44]. Interestingly, the findings of Segarra-Mondejar et al. also suggest that at least a part of the glucose necessary for neurite outgrowth is directly incorporated into newly synthesized lipids [44]. Finally, regional differences in non-oxidative metabolism[26,45] have shown to correlate positively with expression of genes related to synaptic plasticity and development[27]. Taken together, these findings strongly suggest that non-oxidative glucose consumption contributes to biosynthetic processes in the brain. Quantifying the contribution of non-oxidative glucose metabolism to biosynthesis will be an important topic for future studies. Combining a PET marker of protein synthesis[46], such as L-[1-^11^C]-leucine PET[47,48] with measures of non-oxidative glucose use during a learning task could provide further evidence that learning is accompanied by increases in biosynthesis and non-oxidative glucose metabolism. ^13^C magnetic resonance spectroscopy could also be used to measure the movement of glucose and other carbohydrates through different metabolic pathways [49,50].

In summary, on the basis of a meta-analysis of 47 studies, we estimated that non-oxidative processes account for 9% of glucose metabolism in the brain, a significant portion of which cannot be accounted for by lactate efflux to the blood. We also found substantial heterogeneity across studies, likely attributable to differences in methodology. Future studies are needed to determine both the function of non-oxidative metabolism and the ultimate fate of glucose consumed in the brain.

## Methods

### Study design

Our meta-analysis was conducted using the Preferred Reports Items for Systematic Reviews and Meta-Analyses (PRISMA) guidelines[10]. Fig 1 shows a flow diagram of the study procedures. S4 Table contains the PRISMA checklist. We did not complete or register an *a priori* study protocol.

### Eligibility criteria

We included studies that reported mean OGI and/or OCI along with either SD or standard error of the mean (SE), or the data necessary to estimate the mean and SE. Only studies that used arterio-venous differences to measure whole-brain OGI and/or OCI were included. OGI and OGI data were typically taken from text or tables, but were extracted from figures if necessary. S2 Table lists the data source for each study. If a study did not report either ratio but contained the necessary arterio-venous data, we contacted the corresponding author via the listed email address and requested the required data. Although positron emission tomography (PET) can be used to measure whole-brain OGI[51,52], we chose to exclude these studies because of uncertainty in the value of the lumped constant for ^18^F-[FDG][53]. We did not include studies from older adult cohorts or from diseased populations (e.g., cardiac, neurological, or mental disorders).

### Study identification

We searched the PUBMED database with several combinations of the terms “Arterial”, “Arterio”, “Brain”, “Carbohydrate”, “Cerebral”, “Glucose”, “Index”, “OCI”, “OGI”, “Oxygen”, “Ratio”, and “Venous” (S5 Table). In total, we performed 24 separate search queries. All searches were constrained to articles published between 1900 and August 10^th^, 2017. To limit the amount of animal model studies returned by our searches, we added the Medical Subject Heading (MeSH) keyword “Human” to every search. In addition, the first author (TB) conducted a search of his personal archives for any papers that included measures of cerebral oxygen, glucose, and lactate metabolism. The papers in the final dataset that were only found in the first authors archives are listed in S2 Table.

### Statistics

A random effects Bayesian meta-analysis[11] was performed to calculate the population average OGI and OCI. A random effects model accounts for differing variance in each study’s estimates of OGI and OCI, while simultaneously allowing for heterogeneity between studies. Separate models were run for OGI and OCI. If a study reported multiple values for OGI or OCI, a fixed effects meta-analysis was performed to calculate an overall estimate[54]. Our model assumed that each study’s estimate, *y*_*i*_, is a random sample from a normal distribution:

$$y_{i}∼N(\mu +u_{i},\sigma_{i}),$$ (1)

where *μ* is the population mean, *u*_*i*_ is random offset for study *i*, and *σ*_*i*_ is the study standard deviation. No covariates or other explanatory factors were included in the model. We assume that *σ*_*i*_ is equal to each study’s standard error. The random offsets for each study were also assumed to follow a normal distribution:

$$u_{i}∼N(0,\tau ),$$ (2)

where *τ* is the random effects standard deviation, which reflects the heterogeneity across studies.

The model parameters, *μ*, *u*_*i*_, and *τ* were estimated using Hamilton Markov Chain Monte Carlo (MCMC) implemented in Stan[55]. The population mean, *μ*, was given a broad normal prior with a mean of 6 and standard deviation of 2. The random effects standard deviation, *τ*, was given a uniform prior with a lower limit of 0. Eight randomly initialized chains of 20,000 samples were run for each model. The first 10,000 samples of each chain were discarded as warm-up. Sample autocorrelation was minimized by only considering every 5^th^ sample. As a result, all inferences are based upon 16,000 posterior samples. Convergence was assessed using the Gelman and Rubin potential reduction statistic, $\hat{R}$ [56,57]. $\hat{R}$ is the ratio of within chain variance to the pooled between chain variance. At convergence, $\hat{R}$ should be equal to one. For both models, $\hat{R}$ was with within 10^−3^ of 1 for every parameter. All results are summarized with medians and 95% equal-tailed credible intervals.

The primary parameters of interest where the population means, *μ*, for OGI and OCI. We also computed the percent of glucose metabolism that is entirely non-oxidative. This was done by assuming a 6:1 stoichiometric ratio: 100 ∙ (1 − OGI/6.0). Replacing OGI in this expression with OCI gives the percent of carbohydrate metabolism that is non-oxidative.

### Assessment of bias and heterogeneity

Risk of bias within studies was assessed by considering four factors: study population, interval between catheterization and measurement, the presence of experimental manipulations, and fasting state. Bias assessment was not a factor in the random effects meta-analysis, and no sub-group analyses are reported. The possibility for bias across studies was assessed using funnel plots[17]. A funnel plot is used to determine if there is any relationship between the reported OGI/OCI value and its standard error. If a meta-analysis is free from publication bias and heterogeneity, the plot should resemble a funnel with the studies with the smallest standard errors clustered around the population average. An asymmetric funnel plot can be an indication of reporting bias or study heterogeneity[58]. To test for funnel plot asymmetry, we used the method recommended by Egger et al.[17,59], which involves a regression model with effect size as the dependent variable and standard error as the independent variable. Our regression model, implemented in the R metafor package[54], also estimated a random effect for each study.

The possibility of study heterogeneity was further quantified using posterior predictive intervals[60] for a random new study. Posterior predictive intervals, which incorporate the uncertainty in parameter estimates, provide a credible interval in which we would expect a new study to fall. All posterior predictive intervals were computed using 16,000 random samples. Finally, we computed the *I*^2^ statistic[61,62]:

$$I^{2}=100*\frac{{\hat{\tau }}^{2}}{{{\hat{\tau }}^{2}+\hat{\sigma }}^{2}}$$ (3)

where ${\hat{\tau }}^{2}$ is the estimated between study variance from the random effects model, and ${\hat{\sigma }}^{2}$ is the within study variance:

$${\hat{\sigma }}^{2}=\frac{\sum_{i=1}^{k}w_{i}(k-1)}{{\left(\sum_{i=1}^{k}w_{i}\right)}^{2}-\sum_{i=1}^{k}w_{i}^{2}}$$ (4)

where *k* is the number of studies and *w*_*i*_ is the precision of the mean for study *i*: $w_{i}=1/\sigma_{i}^{2}$. We calculated *I*^2^ for each MCMC sample of ${\hat{\tau }}^{2}$ and then computed the median *I*^2^ along with its 95% equal-tailed credible intervals. Higher values of *I*^2^ indicated a greater relative proportion of between study variance and thus greater study heterogeneity.

### Data sharing

All the R scripts and data necessary to reproduce the Figs and analysis in this report can be found at: http://www.github.com/tblazey/ogiMeta.

## Supporting information

S1 Table Papers excluded after a full-text review. (DOCX)

S2 Table Summary characteristics of included studies. Studies that were found only through searching the first authors records are indicated by a *. (NA = Not Applicable).
(DOCX)

S3 Table Assessment of bias within studies. (NA = Not Applicable; NS = Not stated).
(DOCX)

S4 Table PRISMA checklist. (DOCX)

S5 Table PubMed search terms. (DOCX)
