# Supplementary material for: A systematic meta-analysis of oxygen-to-glucose and oxygen-to-carbohydrate ratios in the resting human brain
Source: PLoS One. 2018 Sep 24;13(9):e0204242. doi: 10.1371/journal.pone.0204242 (PMC6152967; doi:10.1371/journal.pone.0204242)
Supplement: S1 Table — (DOCX) [file pone.0204242.s001.docx]

| **Reference** | **PMID** | **Journal** | **Reason for Exclusion** |
| --- | --- | --- | --- |
| **Wortis et al. 1940**[1] | NA | The American journal of psychiatry | Data not sufficient |
| **Gibbs et al. 1945**[2] | NA | The American journal of psychiatry | No control data |
| **Kety et al. 1948a**[3] | 16695569 | The Journal of clinical investigation | Data not sufficient |
| **Kety et al. 1948b**[4] | 16695568 | The Journal of clinical investigation | Data not sufficient |
| **Kety et al. 1951**[5] | 14833874 | Pharmacological reviews | Data not sufficient |
| **Fazekas et al. 1952**[6] | 14902811 | The American journal of the medical sciences | Data not sufficient |
| **Novack et al. 1953**[7] | 13042927 | Circulation | No control data |
| **Scheinberg et al. 1953**[8] | 13057407 | A.M.A. archives of neurology and psychiatry | Data not sufficient |
| **Sokoloff et al. 1953**[9] | 13044828 | The Journal of clinical investigation | Data not sufficient |
| **Schieve et al. 1953**[10] | 13065315 | The American journal of medicine | Data not sufficient |
| **Shenkin et al. 1953**[11] | 13052708 | The Journal of clinical investigation | Data not sufficient |
| **Sokoloff et al. 1955**[12] | 14392225 | The Journal of clinical investigation | Data not sufficient |
| **Mangold et al. 1955**[13] | 14392224 | The Journal of clinical investigation | Data not sufficient |
| **Kety et al. 1956**[14] | 13306754 | Journal of chronic diseases | Data not sufficient |
| **Kennedy et al. 1957**[15] | 13449166 | The Journal of clinical investigation | Data not sufficient |
| **Rowe et al. 1959**[16] | 14439685 | The Journal of clinical investigation | Data not sufficient |
| **Kety et al. 1960**[17] | NA | Methods in Medical Research | Data not sufficient |
| **Porta et al. 1964**[18] | 14127087 | Metabolism: clinical and experimental | No control data |
| **Alexander et al. 1968**[19] | 5635772 | Journal of applied physiology | No control data |
| **Lewis et al. 1974**[20] | 4154353 | Journal of neurochemistry | Data not sufficient |
| **Raichle et al. 1975**[21] | 1155625 | The American journal of physiology | Data not sufficient |
| **Gottstein et al. 1976**[22] | 1271691 | Klinische Wochenschrift | No control data |
| **Van Aken et al. 1976**[23] | 1015217 | Acta anaesthesiologica Belgica | Data not sufficient |
| **Juhlin-Dannfelt et al. 1977**[24] | 929098 | Scandinavian journal of clinical and laboratory investigation | Data not sufficient |
| **Hertz et al. 1981**[25] | 7009645 | The Journal of clinical investigation | No control data |
| **Eriksson et al. 1983**[26] | 6619869 | Journal of neurochemistry | Data not sufficient |
| **Dastur et al. 1985**[27] | 3972914 | Journal of cerebral blood flow and metabolism | Data not sufficient |
| **Fox et al. 1988**[28] | 3260686 | Science | PET study |
| **Hatazawa et al. 1988**[29] | 3259242 | Journal of cerebral blood flow and metabolism | PET study |
| **Warrell et al. 1988**[30] | 2900921 | Lancet | No control data |
| **Blomqvist et al. 1998**[31] | 9789584 | Acta physiologica Scandinavica | Data not sufficient |
| **Grill et al. 1990**[32] | 2110424 | The American journal of physiology | Error of OGI/OCI not reported |
| **Leenders et al. 1990**[33] | 2302536 | Brain | Data not sufficient |
| **Gutniak et al. 1990**[34] | 2185663 | The American journal of physiology | Data not sufficient |
| **Burgess et al. 1991**[35] | 2020274 | Medicine and science in sports and exercise | Data not sufficient |
| **Blomqvist et al. 1991**[36] | 1743207 | European journal of nuclear medicine | Data not sufficient |
| **Pollard et al. 1997**[37] | 9377885 | Critical care medicine | Data not sufficient |
| **Mielck et al. 1998**[38] | 9813515 | British journal of anaesthesia | OGI/OCI not reported |
| **Schaffranietz et al. 1998**[39] | 9584928 | Neurological research | No control data |
| **Wahren et al. 1999**[40] | 10440122 | Diabetologia | OGI/OCI not reported |
| **Madsen et al. 1999**[41] | 10197509 | Journal of cerebral blood flow and metabolism | Data not sufficient |
| **Takahashi et al. 1999**[42] | 10369366 | American journal of neuroradiology | Data not sufficient |
| **Møller et al. 2002**[43] | 12027852 | Acta anaesthesiologica Scandinavica | Repeated data[44] |
| **Nybo et al. 2002**[45] | 12070186 | Journal of applied physiology | Repeated data[46] |
| **Lancaster et al. 2004**[47] | 15544165 | Cell stress & chaperones | Data not sufficient |
| **Dalsgaard et al. 2004**[48] | 15123562 | Experimental physiology | Repeated data[49] |
| **Cremer et al. 2004**[50] | 15114206 | Anesthesiology | Data not sufficient |
| **Blomstrand et al. 2005**[51] | 16218925 | Acta physiologica Scandinavica | Data not sufficient |
| **Chieregato et al. 2007**[52] | 17893572 | Journal of neurosurgical anesthesiology | No control data |
| **Seifert et al. 2008**[53] | 19053964 | Acta physiologica | Data not sufficient |
| **Quistorff et al. 2008**[54] | 18653766 | FASEB journal | Data not sufficient |
| **Holbein et al. 2009**[55] | 19196488 | Critical care | No control data |
| **van Hall et al. 2009**[56] | 19337275 | Journal of cerebral blood flow and metabolism | Data not sufficient |
| **Espenell et al. 2010**[57] | 20661680 | Canadian journal of anesthesia | No control data |
| **Powers et al. 2010**[58] | 20959851 | Journal of cerebral blood flow and metabolism | PET study |
| **Rasmussen et al. 2010**[59] | 20102344 | Acta physiologica | No control data |
| **Smith et al. 2012**[60] | 23019310 | Journal of applied physiology | Data not sufficient |
| **Mikkelsen et al. 2014**[61] | 25415176 | The Journal of clinical endocrinology and metabolism | No control data |
| **Glenn et al. 2015**[62] | 25279664 | Journal of neurotrauma | No control data |
| **Fabricius-Bjerre et al. 2014**[63] | 24903076 | Clinical physiology and functional imaging | No control data |
| **Lewis et al. 2014**[64] | 25217373 | The Journal of physiology | Data not sufficient |
| **Trangmar et al. 2014**[65] | 24835170 | The Journal of physiology | Repeated data[66] |
| **Tholance et al. 2015**[67] | 25668478 | Neurological research | No control data |
| **Slusher et al. 2015**[68] | 25771935 | Journal of neuroendocrinology | Data not sufficient |
| **Grüne et al. 2017**[69] | 28207907 | PloS one | No control data |

**References:**

1. Wortis J, Bowman KM, Goldfarb W. Human Brain Metabolism: Normal Values and Values in Certain Clinical States. Am J Psychiatry. 1940;97: 552–565. doi:10.1176/ajp.97.3.552

2. Gibbs EL, Lennox WG. Bilateral internal jugular blood: Comparison of AV differences, oxygen-dextrose ratios and respiratory quotients. Am J Psychiatry. 1945;: 181–190. doi:10.1176/ajp.102.2.184

3. Kety SS, Schmidt CF. The Effects of Altered Arterial Tensions of Carbon Dioxide and Oxygen on Cerebral Blood Flow and Cerebral Oxygen Consumption of Normal Young Men. J Clin Investig. 1948;27: 484–492. doi:10.1172/JCI101995

4. Seymour S Kety CFS. The Nitrous Oxide Method for the Quantitative Determination of Cerebral Blood Flow in Man: Theory, Procedure and Normal Values. J Clin Investig. 948;27: 476–483. doi:10.1172/JCI101994

5. Kety SS. The theory and applications of the exchange of inert gas at the lungs and tissues. Pharmacol Rev. 1951;3: 1–41.

6. Fazekas JF, Alman RW, Bessman AN. Cerebral physiology of the aged. Am J Med Sci. 1952;223: 245–257.

7. NOVACK P, Goluboff B, Bortin L, SOFFE A, Shenkin HA, Batson P, et al. Studies of the Cerebral Circulation and Metabolism in Congestive Heart Failure. Circulation. 953;7: 724–731. doi:10.1161/01.CIR.7.5.724

8. Scheinberg P, Blackburn I, Rich M, Saslaw M. Effects of aging on cerebral circulation and metabolism. AMA Arch Neurol Psychiatry. 1953;70: 77–85.

9. Sokoloff L, Wechsler RL, Mangold R, Balls K, Kety SS. Cerebral Blood Flow and Oxygen Consumption in Hyperthyroidsim Berfore and After Treatment. J Clin Investig. 953;32: 202–208. doi:10.1172/JCI102728

10. Schieve JF, Wilson WP. The influence of age, anesthesia and cerebral arteriosclerosis on cerebral vascular activity to CO2. The American journal of medicine. 1953;15: 171–174. doi:10.1016/0002-9343(53)90067-9

11. Shenkin HA, Novak P, Goluboff B, Soffe AM, Bortin L, Golden D, et al. The Effects of Aging, Aarteriosclerosis, and Hypertension Upon the Cerebral Circulation. J Clin Investig. 1953;32: 459–465. doi:10.1172/JCI102760

12. Sokoloff L, Mangold R, Wechsler RL, KENNEY C, Kety SS. The effect of mental arithmetic on cerebral circulation and metabolism. J Clin Investig. 1955;34: 1101–1108. doi:10.1172/JCI103159

13. Mangold R, Sokoloff L, Conner E, Kleinerman J, Therman PO, Kety SS. The effects of sleep and lack of sleep on the cerebral circulation and metabolism of normal young men. J Clin Invest. 1955;34: 1092–1100. doi:10.1172/JCI103158

14. Kety SS. Human cerebral blood flow and oxygen consumption as related to aging. J Chronic Dis. 1956;3: 478–486.

15. Kennedy C, Sokoloff L. An adaptation of the nitrous oxide method to the study of the cerebral circulation in children; normal values for cerebral blood flow and cerebral metabolic rate in childhood. J Clin Invest. 1957;36: 1130–1137. doi:10.1172/JCI103509

16. Rowe GG, Maxwell GM, Castillo CA, Freeman DJ, Crumpton CW. A study in man of cerebral blood flow and cerebral glucose, lactate and pyruvate metabolism before and after eating. J Clin Investig. 1959;38: 2154–2158. doi:10.1172/JCI103994

17. Kety SS. Measurement of local blood flow by the exchange of an inert, diffusible substance. Methods Med Res; 1960.

18. Porta PD, Maiolo AT, Negri VU, Rossella E. Cerebral Blood Flow and Metabolism in Therapeutic Insulin Coma. Metab Clin Exp. 1964;13: 131–140. Available: http://eutils.ncbi.nlm.nih.gov/entrez/eutils/elink.fcgi?dbfrom=pubmed&id=14127087&retmode=ref&cmd=prlinks

19. Alexander SC, Smith TC, Strobel G, Stephen GW, Wollman H. Cerebral carbohydrate metabolism of man during respiratory and metabolic alkalosis. J Appl Physiol. 1968;24: 66–72.

20. Lewis LD, Ljunggren B, Norberg K, Siesjö BK. Changes in carbohydrate substrates, amino acids and ammonia in the brain during insulin-induced hypoglycemia. J Neurochem. 1974;23: 659–671. doi:10.1111/j.1471-4159.1974.tb04389.x

21. Raichle ME, Larson KB, Phelps ME, Grubb RL, welch MJ, Ter-Pogossian MM. In vivo measurement of brain glucose transport and metabolism employing glucose-11C. Am J Physiol. 1975;228: 1936–1948.

22. Gottstein U, Zahn U, Held K, Gabriel FH, Textor T, Berghoff W. Effect of hyperventilation on cerebral blood flow and metabolism in man; continuous monitoring of arterio-cerebral venous glucose differences. Klin Wochenschr. 1976;54: 373–381.

23. Van Aken J, Rolly G. Influence of etomidate, a new short acting anesthetic agent, on cerebral blood flow in man. Acta Anaesthesiol Belg. 1976;27 suppl: 175–180.

24. Juhlin-Dannfelt A. Ethanol effects of substrate utilization by the human brain. Scand J Clin Lab Invest. 1977;37: 443–449.

25. Hertz MM, Paulson OB, Barry DI, Christiansen JS, Svendsen PA. Insulin increases glucose transfer across the blood-brain barrier in man. J Clin Investig. 1981;67: 597–604. doi:10.1172/JCI110073

26. Eriksson LS, Law DH, Hagenfeldt L, Wahren J. Nitrogen metabolism of the human brain. J Neurochem. 1983;41: 1324–1328.

27. Dastur DK. Cerebral blood flow and metabolism in normal human aging, pathological aging, and senile dementia. J Cereb Blood Flow Metab. 1985;5: 1–9. doi:10.1038/jcbfm.1985.1

28. Fox PT, Raichle ME, Mintun MA, Dence C. Nonoxidative glucose consumption during focal physiologic neural activity. Science. 1988;241: 462–464.

29. Hatazawa J, Ito M, Matsuzawa T, Ido T, Watanuki S. Measurement of the ratio of cerebral oxygen consumption to glucose utilization by positron emission tomography: its consistency with the values determined by the Kety-Schmidt method in normal volunteers. J Cereb Blood Flow Metab. 1988;8: 426–432. doi:10.1038/jcbfm.1988.79

30. Warrell DA, White NJ, Veall N, Looareesuwan S, Chanthavanich P, Phillips RE, et al. Cerebral anaerobic glycolysis and reduced cerebral oxygen transport in human cerebral malaria. Lancet. 1988;2: 534–538.

31. Blomqvist G, Grill V, Ingvar M, Widén L, Stone-Elander S. The effect of hyperglycaemia on regional cerebral glucose oxidation in humans studied with [1-11C]-D-glucose. Acta Physiol Scand. 1998;163: 403–415. doi:10.1046/j.1365-201X.1998.t01-1-00360.x

32. Grill V, Gutniak M, Bjorkman O, Lindqvist M, Stone-Elander S, Seitz RJ, et al. Cerebral blood flow and substrate utilization in insulin-treated diabetic subjects. Am J Physiol. 1990;258: E813–20. Available: http://eutils.ncbi.nlm.nih.gov/entrez/eutils/elink.fcgi?dbfrom=pubmed&id=2110424&retmode=ref&cmd=prlinks

33. Leenders KL, Perani D, Lammertsma AA, Heather JD, Buckingham P, Healy MJ, et al. Cerebral blood flow, blood volume and oxygen utilization. Normal values and effect of age. Brain. 1990;113 ( Pt 1): 27–47.

34. Gutniak M, Blomqvist G, Widén L, Stone-Elander S, Hamberger B, Grill V. D-[U-11C]glucose uptake and metabolism in the brain of insulin-dependent diabetic subjects. Am J Physiol. 1990;258: E805–12.

35. Burgess ML, Robertson RJ, Davis JM, Norris JM. RPE, blood glucose, and carbohydrate oxidation during exercise: effects of glucose feedings. Med Sci Sports Exerc. 1991;23: 353–359.

36. Blomqvist G, Gjedde A, Gutniak M, Grill V, Widén L, Stone-Elander S, et al. Facilitated transport of glucose from blood to brain in man and the effect of moderate hypoglycaemia on cerebral glucose utilization. Eur J Nucl Med. 1991;18: 834–837.

37. Pollard V, Prough DS, Deyo DJ, Conroy B, Uchida T, Daye A, et al. Cerebral blood flow during experimental endotoxemia in volunteers. Crit Care Med. 1997;25: 1700–1706.

38. Mielck F, Stephan H, Buhre W, Weyland A, Sonntag H. Effects of 1 MAC desflurane on cerebral metabolism, blood flow and carbon dioxide reactivity in humans. Br J Anaesth. 1998;81: 155–160.

39. Schaffranietz L, Heinke W. The effect of different ventilation regimes on jugular venous oxygen saturation in elective neurosurgical patients. Neurological Research. 1998;20 Suppl 1: S66–70. doi:10.1080/01616412.1998.11740613

40. Wahren J, Ekberg K, Fernqvist-Forbes E, Nair S. Brain substrate utilisation during acute hypoglycaemia. Diabetologia. 1999;42: 812–818. Available: http://eutils.ncbi.nlm.nih.gov/entrez/eutils/elink.fcgi?dbfrom=pubmed&id=10440122&retmode=ref&cmd=prlinks

41. Madsen PL, Cruz NF, Sokoloff L, Dienel GA. Cerebral oxygen/glucose ratio is low during sensory stimulation and rises above normal during recovery: excess glucose consumption during stimulation is not accounted for by lactate efflux from or accumulation in brain tissue. J Cereb Blood Flow Metab. 5 ed. 1999;19: 393–400. doi:10.1097/00004647-199904000-00005

42. Takahashi T, Shirane R, Sato S, Yoshimoto T. Developmental changes of cerebral blood flow and oxygen metabolism in children. AJNR Am J Neuroradiol. 1999;20: 917–922.

43. Moller K, Strauss GI, Thomsen G, Larsen FS, Holm S, Sperling BK, et al. Cerebral blood flow, oxidative metabolism and cerebrovascular carbon dioxide reactivity in patients with acute bacterial meningitis. Acta Anaesthesiol Scand. 2002;46: 567–578.

44. Møller K, Strauss GI, Qvist J, Fonsmark L, Knudsen GM, Larsen FS, et al. Cerebral blood flow and oxidative metabolism during human endotoxemia. J Cereb Blood Flow Metab. 2002;22: 1262–1270. doi:10.1097/01.WCB.0000037999.34930.CA

45. Nybo L, Møller K, Volianitis S, Nielsen B, Secher NH. Effects of hyperthermia on cerebral blood flow and metabolism during prolonged exercise in humans. J Appl Physiol. 2002;93: 58–64. doi:10.1152/japplphysiol.00049.2002

46. Nybo L, Nielsen B, Blomstrand E, Møller K, Secher N. Neurohumoral responses during prolonged exercise in humans. J Appl Physiol. 2003;95: 1125–1131. doi:10.1152/japplphysiol.00241.2003

47. Lancaster GI, Moller K, Nielsen B, Secher NH, Febbraio MA, Nybo L. Exercise induces the release of heat shock protein 72 from the human brain in vivo. Cell Stress Chaperones. 2004;9: 276–280. doi:10.1379/CSC-18R.1

48. Dalsgaard MK, Ott P, Dela F, Juul A, Pedersen BK, Warberg J, et al. The CSF and arterial to internal jugular venous hormonal differences during exercise in humans. Exp Physiol. 2004;89: 271–277. doi:10.1113/expphysiol.2003.026922

49. Dalsgaard MK, Quistorff B, Danielsen ER, Selmer C, Vogelsang T, Secher NH. A reduced cerebral metabolic ratio in exercise reflects metabolism and not accumulation of lactate within the human brain. J Physiol (Lond). 2004;554: 571–578. doi:10.1113/jphysiol.2003.055053

50. Cremer OL, Diephuis JC, van Soest H, Vaessen PHB, Bruens MGJ, Hennis PJ, et al. Cerebral oxygen extraction and autoregulation during extracorporeal whole body hyperthermia in humans. Anesthesiology. 2004;100: 1101–1107.

51. Blomstrand E, Moller K, Secher NH, Nybo L. Effect of carbohydrate ingestion on brain exchange of amino acids during sustained exercise in human subjects. Acta Physiol Scand. 2005;185: 203–209. doi:10.1111/j.1365-201X.2005.01482.x

52. Chieregato A, Marchi M, Fainardi E, Targa L. Cerebral Arterio-venous pCO2 Difference, Estimated Respiratory Quotient, and Early Posttraumatic Outcome: Comparison With Arterio-venous Lactate and Oxygen Differences. Journal of Neurosurgical Anesthesiology. 2007;19: 222–228. doi:10.1097/ANA.0b013e31806589f6

53. Seifert T, Rasmussen P, Secher NH, Nielsen HB. Cerebral oxygenation decreases during exercise in humans with beta-adrenergic blockade. Acta Physiol (Oxf). 2009;196: 295–302. doi:10.1111/j.1748-1716.2008.01946.x

54. Quistorff B, Secher NH, van Lieshout JJ. Lactate fuels the human brain during exercise. FASEB J. 2008;22: 3443–3449. doi:10.1096/fj.08-106104

55. Holbein M, Béchir M, Ludwig S, Sommerfeld J, Cottini SR, Keel M, et al. Differential influence of arterial blood glucose on cerebral metabolism following severe traumatic brain injury. Crit Care. 2009;13: R13. doi:10.1186/cc7711

56. van Hall G, Strømstad M, Rasmussen P, Jans O, Zaar M, Gam C, et al. Blood lactate is an important energy source for the human brain. J Cereb Blood Flow Metab. 3rd ed. 2009;29: 1121–1129. doi:10.1038/jcbfm.2009.35

57. Espenell AEG, McIntyre IW, Gulati H, Girling LG, Wilkinson MF, Silvaggio JA, et al. Lactate flux during carotid endarterectomy under general anesthesia: correlation with various point-of-care monitors. Can J Anaesth. 2010;57: 903–912. doi:10.1007/s12630-010-9356-7

58. Powers WJ, Haas RH, Le T, Videen TO, Markham J, Perlmutter JS. Platelet mitochondrial complex I and I+III activities do not correlate with cerebral mitochondrial oxidative metabolism. J Cereb Blood Flow Metab. 2011;31: e1–5. doi:10.1038/jcbfm.2010.179

59. Rasmussen P, Nybo L, Volianitis S, Moller K, Secher NH, Gjedde A. Cerebral oxygenation is reduced during hyperthermic exercise in humans. Acta Physiol (Oxf). 2010;199: 63–70. doi:10.1111/j.1748-1716.2010.02084.x

60. Smith ZM, Krizay E, Guo J, Shin DD, Scadeng M, Dubowitz DJ. Sustained high-altitude hypoxia increases cerebral oxygen metabolism. J Appl Physiol. 2013;114: 11–18. doi:10.1152/japplphysiol.00703.2012

61. Mikkelsen KH, Seifert T, Secher NH, Grøndal T, van Hall G. Systemic, Cerebral and Skeletal Muscle Ketone Body and Energy Metabolism During Acute Hyper-D-β-Hydroxybutyratemia in Post-Absorptive Healthy Males. J Clin Endocrinol Metab. 2015;100: 636–643. doi:10.1210/jc.2014-2608

62. Glenn TC, Martin NA, McArthur DL, Hovda DA, Vespa P, Johnson ML, et al. Endogenous Nutritive Support after Traumatic Brain Injury: Peripheral Lactate Production for Glucose Supply via Gluconeogenesis. J Neurotrauma. 2015;32: 811–819. doi:10.1089/neu.2014.3482

63. Fabricius-Bjerre A, Overgaard A, Winther-Olesen M, Lönn L, Secher NH, Nielsen HB. Reduced cerebral oxygen-carbohydrate index during endotracheal intubation in vascular surgical patients. Clin Physiol Funct Imaging. 2015;35: 404–410. doi:10.1111/cpf.12176

64. Lewis NCS, Bain AR, MacLeod DB, Wildfong KW, Smith KJ, Willie CK, et al. Impact of hypocapnia and cerebral perfusion on orthostatic tolerance. J Physiol (Lond). 2014;592: 5203–5219. doi:10.1113/jphysiol.2014.280586

65. Trangmar SJ, Chiesa ST, Stock CG, Kalsi KK, Secher NH, González-Alonso J. Dehydration affects cerebral blood flow but not its metabolic rate for oxygen during maximal exercise in trained humans. J Physiol (Lond). 2014;592: 3143–3160. doi:10.1113/jphysiol.2014.272104

66. Trangmar SJ, Chiesa ST, Llodio I, Garcia B, Kalsi KK, Secher NH, et al. Dehydration accelerates reductions in cerebral blood flow during prolonged exercise in the heat without compromising brain metabolism. Am J Physiol Heart Circ Physiol. 2015;309: H1598–607. doi:10.1152/ajpheart.00525.2015

67. Tholance Y, Barcelos GK, Dailler F, Renaud B, Marinesco S, Perret-Liaudet A. Biochemical neuromonitoring of poor-grade aneurysmal subarachnoid hemorrhage: comparative analysis of metabolic events detected by cerebral microdialysis and by retrograde jugular vein catheterization. Neurological Research. 2015;37: 578–587. doi:10.1179/1743132815Y.0000000012

68. Slusher AL, Whitehurst M, Zoeller RF, Mock JT, Maharaj A, Huang CJ. Brain-Derived Neurotrophic Factor and Substrate Utilization Following Acute Aerobic Exercise in Obese Individuals. J Neuroendocrinol. 2015;27: 370–376. doi:10.1111/jne.12275

69. Grüne F, Kazmaier S, Hoeks SE, Stolker RJ, Coburn M, Weyland A. Argon does not affect cerebral circulation or metabolism in male humans. PLoS ONE. 2017;12: e0171962. doi:10.1371/journal.pone.0171962
