# Supplementary material for: A systematic meta-analysis of oxygen-to-glucose and oxygen-to-carbohydrate ratios in the resting human brain
Source: PLoS One. 2018 Sep 24;13(9):e0204242. doi: 10.1371/journal.pone.0204242 (PMC6152967; doi:10.1371/journal.pone.0204242)
Supplement: S3 Table — (NA = Not Applicable; NS = Not stated). (DOCX) [file pone.0204242.s003.docx]

| **Reference** | **Study Population** | **Waiting Period** | **Position** | **Experimental Manipulation(s)** | **Fasting** |
| --- | --- | --- | --- | --- | --- |
| **Gibbs et al., 1942**[1] | All males. | ~30 min. | Supine | None | NS |
| **Scheinberg et al., 1949**[2] | 3 illness free hospital patients. | 40 to 50 min. | Supine and Erect | None | Yes |
| **Sokoloff et al., 1957**[3] | 1 participant was a patient with an anxiety disorder. | NS | NS | Some participants had been given LSD a week earlier. | NS |
| **Eisenberg et al., 1962**[4] | None | NS | NS | None | Yes |
| **Dastur et al., 1963**[5] | None | NS | NS | None | NS |
| **Gottstein et al., 1963**[6] | Patients aged up to 61 years | NS | NS | None | NS |
| **Scheinberg et al., 1965**[7] | Some subjects were hospital patients without disease | 20 min. | Supine | None | Yes (12 hours) |
| **Cohen et al., 1967**[8] | All male | NS | Supine | None | Yes |
| **Gottstein et al., 1967**[9] | Patients with normal metabolism | NS | NS | None | NS |
| **Raichle et al., 1970**[10] | None | NS | Reclining | None | NS |
| **Takeshita, 1972**[11] | Patients scheduled for elective surgery | NS | NS | Given atropine | NS |
| **Blomqvist et al., 1990**[12] | None | NS | NS | None | NS |
| **Boyle et al., 1994**[13] | None | 2 hours | NS | Sleep reduction; Subjects were infused with 6,6-D2-glucose; | No |
| **Madsen et al., 1995**[14] | None | ~1 hour | Supine | None | Yes (14 hours) |
| **Hasselbalch et al., 1996**[15] | None | 1 hour | Supine | Saline infusion | No |
| **Ide et al., 1999**[16] | None | NS | Semi-supine | None | No |
| **Ide et al., 2000**[17] | None | NS | Semi-supine | None | NS |
| **Dalsgaard et al., 2002**[18] | None | NS | Semi-supine | None | NS |
| **Møller et al., 2002**[19] | None | 1 hour | Supine | Subjects were infused with isotonic glucose | Yes (Overnight) |
| **Dalsgaard et al., 2003**[20] | None | NS | Semi-supine | None | No |
| **Glenn et al., 2003**[21] | None | NS | NS | None | NS |
| **Nybo et al., 2003**[22] | All trained males | 30 min. | NS | None | No |
| **Strauss et al., 2003**[23] | None | NS | Supine | Subjects were infused with 5% glucose. | Yes (Overnight) |
| **Dalsgaard et al., 2004a**[24] | None | NS | Semi-supine | None | Yes (8 hours) |
| **Dalsgaard et al., 2004b**[25] | All male | 1 hour | Semi-supine | None | Yes (Overnight) |
| **Dalsgaard et al., 2004c**[26] | All male | NS | Semi-supine | None | Yes (Overnight) |
| **Ogoh et al., 2005**[27] | All male | NS | Semi-supine | None | Yes (Overnight) |
| **Rasmussen et al., 2006**[28] | All male | 1.5 hours | Semi-supine | None | Yes (Overnight) |
| **Larsen et al., 2008**[29] | All male | 1 hour | Semi-supine | None | Yes (Overnight) |
| **Voliantis et al., 2008**[30] | All trained male rowers | NS | Semi-supine | None | Yes (8 hours) |
| **Bailey et al., 2009**[31] | All male | 30 min. | Supine | Subjects breathed 21% O_2_ | Yes (12 hours) |
| **Gam et al., 2009**[32] | All male | 1 hour | Semi-supine | None | NS |
| **Seifert et al., 2009a**[33] | All overweight males | 1 hour | Semi-supine | None | No |
| **Seifert et al., 2009b**[34] | None | 1 hour | Supine | Some subjects received a saline control infusion | No |
| **Rasmussen et al, 2010a**[35] | All male | NS | Semi-supine | None | NS |
| **Rasmussen et al, 2010b**[36] | All male | NS | Semi-supine | Saline injected control; Some had received an EPO trial 3 months earlier | NS |
| **Seifert et al., 2010**[37] | All male | 30 min. | Semi-supine | None | NS |
| **Volianitis et al., 2011**[38] | All males and competitive rowers | NS | Semi-supine | Saline infusion control; Some subjects performed bicarbonate trial ~1-2 weeks earlier | Yes (Overnight) |
| **Overgaard et al., 2012**[39] | None | 2 hours | Supine | Some subjects performed a hypoxia exercise trial at least two weeks prior; Infused with [1-^13^C] lactate and labeled bicarbonate | No |
| **Fisher et al., 2013**[40] | None | 1 hour | Semi-supine | None | Yes (2h) |
| **Ainslie et al., 2014**[41] | None | 30 min. | Supine | Some measurements were performed after hypoxia trials. | Yes (2h) |
| **Smith et al., 2014**[42] | All male | 25 min. | Supine | None | Yes (4h) |
| **Glenn et al., 2015**[43] | None | NS | Semi-supine | Infusion of [3-^13^C] lactate and D2 glucose | NS |
| **Trangmar et al., 2015**[44] | All trained males | ~1 hour | Semi-supine | None | NS |
| **Willie et al., 2015**[45] | All male | ~30 min. | NS | Subjects underwent an arterial blood gas clamp | NS |
| **Bain et al., 2016**[46] | All subjects were elite breath hold divers | 30 min | NS | None | NS |
| **Bain et al., 2017**[47] | All subjects were elite breath hold divers | NS | NS | None | NS |

**References:**

1. Gibbs EL, Lennox WG, Nims LF, Gibbs FA. Arterial and cerebral venous blood arterial-venous differences in man. J Biol Chem. Journal of Biological Chemistry; 1942;144: 325–332.

2. Scheinberg P, Stead EA. The Cerebral Blood Flow In Male Subjects As Measured By The Nitrous Oxide Technique. J Clin Investig. 1949;28: 1163–1171. doi:10.1172/JCI102150

3. Sokoloff L, Perlin S, Kornetsky C, Kety SS. The effects of D-lysergic acid diethylamide on cerebral circulation and overall metabolism. Ann N Y Acad Sci. 1957;66: 468–477.

4. Eisenberg S, Seltzer HS. Cerebral Metabolic Effects of Acutely Induced Hypoglycemia in Human Subjects. Metabolism. 1962;11: 1162–1168.

5. Dastur DK, Lane MH, Hansen DB, Kety SS, Butler RN, Perlin S, et al. Effects of aging on cerebral circulation and metabolism in man. In: JE B, Butler RN, Greenhouse SW, Sokoloff L, Yarrow MR, editors. Human Aging: A Biological and Behavioral Study. Bethesda, US. 1963. pp. 59–76.

6. Gottstein U, Bernsmeier A, Sedlmeyer I. The Carbohydrate Metabolism Of The Human Brain. Klin Wochenschr. 1963;41: 943–948.

7. Scheinberg P, Bourne B, Reinmuth OM. Human Cerebral Lactate And Pyruvate Extraction. Arch Neurol. 1965;12: 246–250.

8. Cohen PJ, Alexander SC, Smith TC, Reivich M, Wollman H. Effects of hypoxia and normocarbia on cerebral blood flow and metabolism in conscious man. J Appl Physiol. 1967;23: 183–189.

9. Gottstein U, Held K. The effect of insulin on brain metabolism in metabolically healthy and diabetic patients. Klin Wochenschr. 1967;45: 18–23. doi:10.1007/BF01745733

10. Raichle ME, Posner JB, Plum F. Cerebral blood flow during and after hyperventilation. Arch Neurol. 1970;23: 394–403. doi:10.1001/archneur.1970.00480290014002

11. Takeshita H, Okuda Y, Sari A. The effects of ketamine on cerebral circulation and metabolism in man. Anesthesiology. 1972;36: 69–75.

12. Blomqvist G, Stone-Elander S, Halldin C, Roland PE, Widén L, Lindqvist M, et al. Positron emission tomographic measurements of cerebral glucose utilization using [1-11C]D-glucose. J Cereb Blood Flow Metab. 1990;10: 467–483. doi:10.1038/jcbfm.1990.89

13. Boyle PJ, Scott JC, Krentz AJ, Nagy RJ, Comstock E, Hoffman C. Diminished brain glucose metabolism is a significant determinant for falling rates of systemic glucose utilization during sleep in normal humans. J Clin Invest. 1994;93: 529–535. doi:10.1172/JCI117003

14. Madsen PL, Hasselbalch SG, Hagemann LP, Olsen KS, Bülow J, Holm S, et al. Persistent resetting of the cerebral oxygen/glucose uptake ratio by brain activation: evidence obtained with the Kety-Schmidt technique. J Cereb Blood Flow Metab. 1995;15: 485–491. doi:10.1038/jcbfm.1995.60

15. Hasselbalch SG, Madsen PL, Hageman LP, Olsen KS, Justesen N, Holm S, et al. Changes in cerebral blood flow and carbohydrate metabolism during acute hyperketonemia. Am J Physiol. 1996;270: E746–51.

16. Ide K, Horn A, Secher NH. Cerebral metabolic response to submaximal exercise. J Appl Physiol. 1999;87: 1604–1608. doi:10.1152/japplphysiol.00534.2017

17. Ide K, Schmalbruch IK, Quistorff B, Horn A, Secher NH. Lactate, glucose and O2 uptake in human brain during recovery from maximal exercise. J Physiol (Lond). 2000;522 Pt 1: 159–164. doi:10.1111/j.1469-7793.2000.t01-2-00159.xm

18. Dalsgaard MK, Ide K, Cai Y, Quistorff B, Secher NH. The intent to exercise influences the cerebral O(2)/carbohydrate uptake ratio in humans. J Physiol (Lond). 2002;540: 681–689. doi:10.1113/jphysiol.2001.013062

19. Møller K, Strauss GI, Qvist J, Fonsmark L, Knudsen GM, Larsen FS, et al. Cerebral blood flow and oxidative metabolism during human endotoxemia. J Cereb Blood Flow Metab. 2002;22: 1262–1270. doi:10.1097/01.WCB.0000037999.34930.CA

20. Dalsgaard MK, Nybo L, Cai Y, Secher NH. Cerebral metabolism is influenced by muscle ischaemia during exercise in humans. Exp Physiol. 2003;88: 297–302. doi:10.1113/eph8802469

21. Glenn TC, Kelly DF, Boscardin WJ, McArthur DL, Vespa P, Oertel M, et al. Energy dysfunction as a predictor of outcome after moderate or severe head injury: indices of oxygen, glucose, and lactate metabolism. J Cereb Blood Flow Metab. 2003;23: 1239–1250. doi:10.1097/01.WCB.0000089833.23606.7F

22. Nybo L, Nielsen B, Blomstrand E, Møller K, Secher N. Neurohumoral responses during prolonged exercise in humans. J Appl Physiol. 2003;95: 1125–1131. doi:10.1152/japplphysiol.00241.2003

23. Strauss GI, Møller K, Larsen FS, Kondrup J, Knudsen GM. Cerebral glucose and oxygen metabolism in patients with fulminant hepatic failure. Liver Transpl. 2003;9: 1244–1252. doi:10.1016/j.lts.2003.09.020

24. Dalsgaard MK, Quistorff B, Danielsen ER, Selmer C, Vogelsang T, Secher NH. A reduced cerebral metabolic ratio in exercise reflects metabolism and not accumulation of lactate within the human brain. J Physiol (Lond). 2004;554: 571–578. doi:10.1113/jphysiol.2003.055053

25. Dalsgaard MK, Ogoh S, Dawson EA, Yoshiga CC, Quistorff B, Secher NH. Cerebral carbohydrate cost of physical exertion in humans. Am J Physiol Regul Integr Comp Physiol. 2004;287: R534–40. doi:10.1152/ajpregu.00256.2004

26. Dalsgaard MK, Volianitis S, Yoshiga CC, Dawson EA, Secher NH. Cerebral metabolism during upper and lower body exercise. J Appl Physiol. 2004;97: 1733–1739. doi:10.1152/japplphysiol.00450.2004

27. Ogoh S, Dalsgaard MK, Yoshiga CC, Dawson EA, Keller DM, Raven PB, et al. Dynamic cerebral autoregulation during exhaustive exercise in humans. Am J Physiol Heart Circ Physiol. 2005;288: H1461–7. doi:10.1152/ajpheart.00948.2004

28. Rasmussen P, Plomgaard P, Krogh-Madsen R, Kim Y-S, van Lieshout JJ, Secher NH, et al. MCA Vmean and the arterial lactate-to-pyruvate ratio correlate during rhythmic handgrip. J Appl Physiol. 2006;101: 1406–1411. doi:10.1152/japplphysiol.00423.2006

29. Larsen TS, Rasmussen P, Overgaard M, Secher NH, Nielsen HB. Non-selective beta-adrenergic blockade prevents reduction of the cerebral metabolic ratio during exhaustive exercise in humans. J Physiol (Lond). 2008;586: 2807–2815. doi:10.1113/jphysiol.2008.151449

30. Volianitis S, Fabricius-Bjerre A, Overgaard A, Strømstad M, Bjarrum M, Carlson C, et al. The cerebral metabolic ratio is not affected by oxygen availability during maximal exercise in humans. J Physiol (Lond). 2008;586: 107–112. doi:10.1113/jphysiol.2007.142273

31. Bailey DM, Taudorf S, Berg RMG, Lundby C, McEneny J, Young IS, et al. Increased cerebral output of free radicals during hypoxia: implications for acute mountain sickness? Am J Physiol Regul Integr Comp Physiol. 2009;297: R1283–92. doi:10.1152/ajpregu.00366.2009

32. Gam CMB, Rasmussen P, Secher NH, Seifert T, Larsen FS, Nielsen HB. Maintained cerebral metabolic ratio during exercise in patients with beta-adrenergic blockade. Clin Physiol Funct Imaging. 2009;29: 420–426. doi:10.1111/j.1475-097X.2009.00889.x

33. Seifert T, Rasmussen P, Brassard P, Homann PH, Wissenberg M, Nordby P, et al. Cerebral oxygenation and metabolism during exercise following three months of endurance training in healthy overweight males. Am J Physiol Regul Integr Comp Physiol. 2009;297: R867–76. doi:10.1152/ajpregu.00277.2009

34. Seifert TS, Brassard P, Jørgensen TB, Hamada AJ, Rasmussen P, Quistorff B, et al. Cerebral non-oxidative carbohydrate consumption in humans driven by adrenaline. J Physiol (Lond). 2009;587: 285–293. doi:10.1113/jphysiol.2008.162073

35. Rasmussen P, Nielsen J, Overgaard M, Krogh-Madsen R, Gjedde A, Secher NH, et al. Reduced muscle activation during exercise related to brain oxygenation and metabolism in humans. J Physiol (Lond). 2010;588: 1985–1995. doi:10.1113/jphysiol.2009.186767

36. Rasmussen P, Foged EM, Krogh-Madsen R, Nielsen J, Nielsen TR, Olsen NV, et al. Effects of erythropoietin administration on cerebral metabolism and exercise capacity in men. J Appl Physiol. 2010;109: 476–483. doi:10.1152/japplphysiol.00234.2010

37. Seifert T, Fisher JP, Young CN, Hartwich D, Ogoh S, Raven PB, et al. Glycopyrrolate abolishes the exercise-induced increase in cerebral perfusion in humans. Exp Physiol. 2010;95: 1016–1025. doi:10.1113/expphysiol.2010.054346

38. Volianitis S, Rasmussen P, Seifert T, Nielsen HB, Secher NH. Plasma pH does not influence the cerebral metabolic ratio during maximal whole body exercise. J Physiol (Lond). 2011;589: 423–429. doi:10.1113/jphysiol.2010.195636

39. Overgaard M, Rasmussen P, Bohm AM, Seifert T, Brassard P, Zaar M, et al. Hypoxia and exercise provoke both lactate release and lactate oxidation by the human brain. FASEB J. 2012;26: 3012–3020. doi:10.1096/fj.11-191999

40. Fisher JP, Hartwich D, Seifert T, Olesen ND, McNulty CL, Nielsen HB, et al. Cerebral perfusion, oxygenation and metabolism during exercise in young and elderly individuals. J Physiol (Lond). 2013;591: 1859–1870. doi:10.1113/jphysiol.2012.244905

41. Ainslie PN, Shaw AD, Smith KJ, Willie CK, Ikeda K, Graham J, et al. Stability of cerebral metabolism and substrate availability in humans during hypoxia and hyperoxia. 2014;126: 661–670. doi:10.1042/CS20130343

42. Smith KJ, MacLeod D, Willie CK, Lewis NCS, Hoiland RL, Ikeda K, et al. Influence of high altitude on cerebral blood flow and fuel utilization during exercise and recovery. J Physiol (Lond). 2014;592: 5507–5527. doi:10.1113/jphysiol.2014.281212

43. Glenn TC, Martin NA, Horning MA, McArthur DL, Hovda DA, Vespa P, et al. Lactate: brain fuel in human traumatic brain injury: a comparison with normal healthy control subjects. J Neurotrauma. 2015;32: 820–832. doi:10.1089/neu.2014.3483

44. Trangmar SJ, Chiesa ST, Llodio I, Garcia B, Kalsi KK, Secher NH, et al. Dehydration accelerates reductions in cerebral blood flow during prolonged exercise in the heat without compromising brain metabolism. Am J Physiol Heart Circ Physiol. 2015;309: H1598–607. doi:10.1152/ajpheart.00525.2015

45. Willie CK, MacLeod DB, Smith KJ, Lewis NC, Foster GE, Ikeda K, et al. The contribution of arterial blood gases in cerebral blood flow regulation and fuel utilization in man at high altitude. J Cereb Blood Flow Metab. 2015;35: 873–881. doi:10.1038/jcbfm.2015.4

46. Bain AR, Ainslie PN, Hoiland RL, Barak OF, Cavar M, Drvis I, et al. Cerebral oxidative metabolism is decreased with extreme apnoea in humans; impact of hypercapnia. J Physiol (Lond). 2016;594: 5317–5328. doi:10.1113/JP272404

47. Bain AR, Ainslie PN, Barak OF, Hoiland RL, Drvis I, Mijacika T, et al. Hypercapnia is essential to reduce the cerebral oxidative metabolism during extreme apnea in humans. J Cereb Blood Flow Metab. 2017;37: 3231–3242. doi:10.1177/0271678X16686093
