# Supplementary material for: A systematic meta-analysis of oxygen-to-glucose and oxygen-to-carbohydrate ratios in the resting human brain
Source: PLoS One. 2018 Sep 24;13(9):e0204242. doi: 10.1371/journal.pone.0204242 (PMC6152967; doi:10.1371/journal.pone.0204242)
Supplement: S5 Table — (DOCX) [file pone.0204242.s005.docx]

| **Number** | **Terms** |
| --- | --- |
| 1 | Oxygen AND Arterio AND Venous AND Brain |
| 2 | Oxygen AND Arterio AND Venous AND Cerebral |
| 3 | Lactate AND Arterio AND Venous AND Brain |
| 4 | Lactate AND Arterio AND Venous AND Cerebral |
| 5 | Glucose AND Arterio AND Venous AND Brain |
| 6 | Glucose AND Arterio AND Venous AND Cerebral |
| 7 | Oxygen AND Arterial AND Venous AND Brain |
| 8 | Oxygen AND Arterial AND Venous AND Cerebral |
| 9 | Lactate AND Arterial AND Venous AND Brain |
| 10 | Lactate AND Arterial AND Venous AND Cerebral |
| 11 | Glucose AND Arterial AND Venous AND Brain |
| 12 | Glucose AND Arterial AND Venous AND Cerebral |
| 13 | OGI AND Brain |
| 14 | OGI AND Cerebral |
| 15 | OCI AND Brain |
| 16 | OCI AND Cerebral |
| 17 | Oxygen AND Glucose AND Index AND Brain |
| 18 | Oxygen AND Glucose AND Index AND Cerebral |
| 19 | Oxygen AND Carbohydrate AND Index AND Brain |
| 20 | Oxygen AND Carbohydrate AND Index AND Cerebral |
| 21 | Oxygen AND Glucose AND Ratio AND Brain |
| 22 | Oxygen AND Glucose AND Ratio AND Cerebral |
| 23 | Oxygen AND Carbohydrate AND Ratio AND Brain |
| 24 | Oxygen AND Carbohydrate AND Ratio AND Cerebral |
